# Successful bridge to recovery in fulminant myocarditis using a biventricular assist device: a case report

**DOI:** 10.1186/s13256-017-1466-1

**Published:** 2017-10-24

**Authors:** Yusuke Adachi, Osamu Kinoshita, Masaru Hatano, Yukako Shintani, Noritsugu Naito, Mitsutoshi Kimura, Kan Nawata, Daisuke Nitta, Hisataka Maki, Kazutaka Ueda, Eisuke Amiya, Eiki Takimoto, Issei Komuro, Minoru Ono

**Affiliations:** 1 Department of Cardiovascular Medicine, Graduate School of Medicine, The University of Tokyo, Tokyo, Japan; 2 Department of Cardiac Surgery, Graduate School of Medicine, The University of Tokyo, 7-3-1, Hongo, Bunkyo-ku, Tokyo, 113-8655 Japan; 3 Department of Therapeutic Strategy for Heart Failure, Graduate School of Medicine, The University of Tokyo, Tokyo, Japan; 4 Department of Pathology, Graduate School of Medicine, The University of Tokyo, Tokyo, Japan; 5 Department of Ubiquitous Preventive Medicine, Graduate School of Medicine, The University of Tokyo, Tokyo, Japan; 6 Department of Advanced Translational Research and Medicine in Management of Pulmonary Hypertension, Graduate School of Medicine, The University of Tokyo, Tokyo, Japan

**Keywords:** Peripheral venoarterial extracorporeal membrane oxygenation, Fulminant myocarditis, Ventricular assist device, Biventricular assist device, Right heart failure, Case report

## Abstract

**Background:**

Fulminant myocarditis is a life-threatening disease, and myocardial damage expands the right ventricle as well as the left ventricle in some cases. There is a mortality rate of over 40% in patients with fulminant myocarditis who need mechanical circulatory support by peripheral venoarterial extracorporeal membrane oxygenation.

**Case presentation:**

We report a case of a 27-year-old Japanese woman who was successfully bridged to recovery by using a biventricular assist device. She was diagnosed with fulminant myocarditis, and peripheral venoarterial extracorporeal membrane oxygenation was established on the same day. Her left ventricular ejection fraction rapidly decreased from 40% to 5% in 3 days and weaning from venoarterial extracorporeal membrane oxygenation was deemed difficult. Therefore, we performed a ventricular assist device implantation on day 4. A left ventricular assist device was implanted first. However, adequate blood flow did not circulate to the left side of her heart because of right-sided heart failure. Thus, an additional implant of a right ventricular assist device was performed during the operation. Her left ventricular ejection fraction recovered to 50% on day 10. The biventricular assist device was successfully removed on day 14. She has not experienced worsening of biventricular function during her follow-ups for 4 years.

**Conclusions:**

Ventricular assist device therapy should be considered if there is no improvement in cardiac function in patients with fulminant myocarditis regardless of several days of support by venoarterial extracorporeal membrane oxygenation. A right ventricular assist device should always be implemented when necessary because biventricular involvement is not uncommon in fulminant myocarditis.

**Electronic supplementary material:**

The online version of this article (doi:10.1186/s13256-017-1466-1) contains supplementary material, which is available to authorized users.

## Background

Fulminant myocarditis is often life-threatening because it may progress to cardiogenic shock. Peripheral venoarterial extracorporeal membrane oxygenation (VA-ECMO) is indicated for low output syndrome or life-threatening arrhythmia caused by fulminant myocarditis. A national survey in Japan showed that the mortality of patients with fulminant myocarditis who needed VA-ECMO was over 40% [1]. VA-ECMO is a life-saving device and should be immediately considered in the case of advanced cardiogenic shock caused by fulminant myocarditis. However, there may be potential complications, such as multiple organ failure due to insufficient blood perfusion and pulmonary edema due to incomplete left ventricular unloading. It is important to understand whether other devices, such as a ventricular assist device (VAD), should be implemented in the case of insufficient circulatory support by VA-ECMO. We report a case of fulminant myocarditis successfully bridged to recovery by changing from a VA-ECMO to a biventricular assist device (BiVAD).

## Case presentation

A 27-year-old previously healthy Japanese woman presented to a local hospital with chest discomfort and vomiting (day 1). She had no family history of cardiovascular disease, was a social drinker, and had never smoked. Her systolic blood pressure was 70 mmHg, a cardiac troponin T rapid test gave a positive result, and an electrocardiogram (ECG) showed an ST elevation in broad precordial leads. Transthoracic echocardiography showed edematous left ventricular myocardium and a left ventricular ejection fraction (LVEF) of 40% with diffuse hypokinetic wall motion. Myocarditis was suspected, and cardiac catheterization was performed after intubation and the insertion of an intra-aortic balloon pump (IABP). Coronary angiography revealed no coronary artery stenosis and left ventriculography showed diffuse hypokinesis (Additional file 1: Movie 1). A right ventricular endomyocardial biopsy was performed at the same time. After catheterization, ventricular tachycardia suddenly appeared, her hemodynamics became unstable, and VA-ECMO was immediately employed (day 1). Regardless of ECMO support, her LVEF continued to decrease to 20% on day 2 and to 10% on day 3 (Additional file 2: Movie 2). She was transferred to our hospital on day 3.


Additional file 1: Movie 1. Left ventriculography on day 1 showed diffuse hypokinesis. (WMV 1201 kb)



Additional file 2: Movie 2. Transthoracic echocardiography on day 3 showed that the patient's left ventricular ejection fraction decreased to 10%. (WMV 427 kb)


**Fig. 1:**
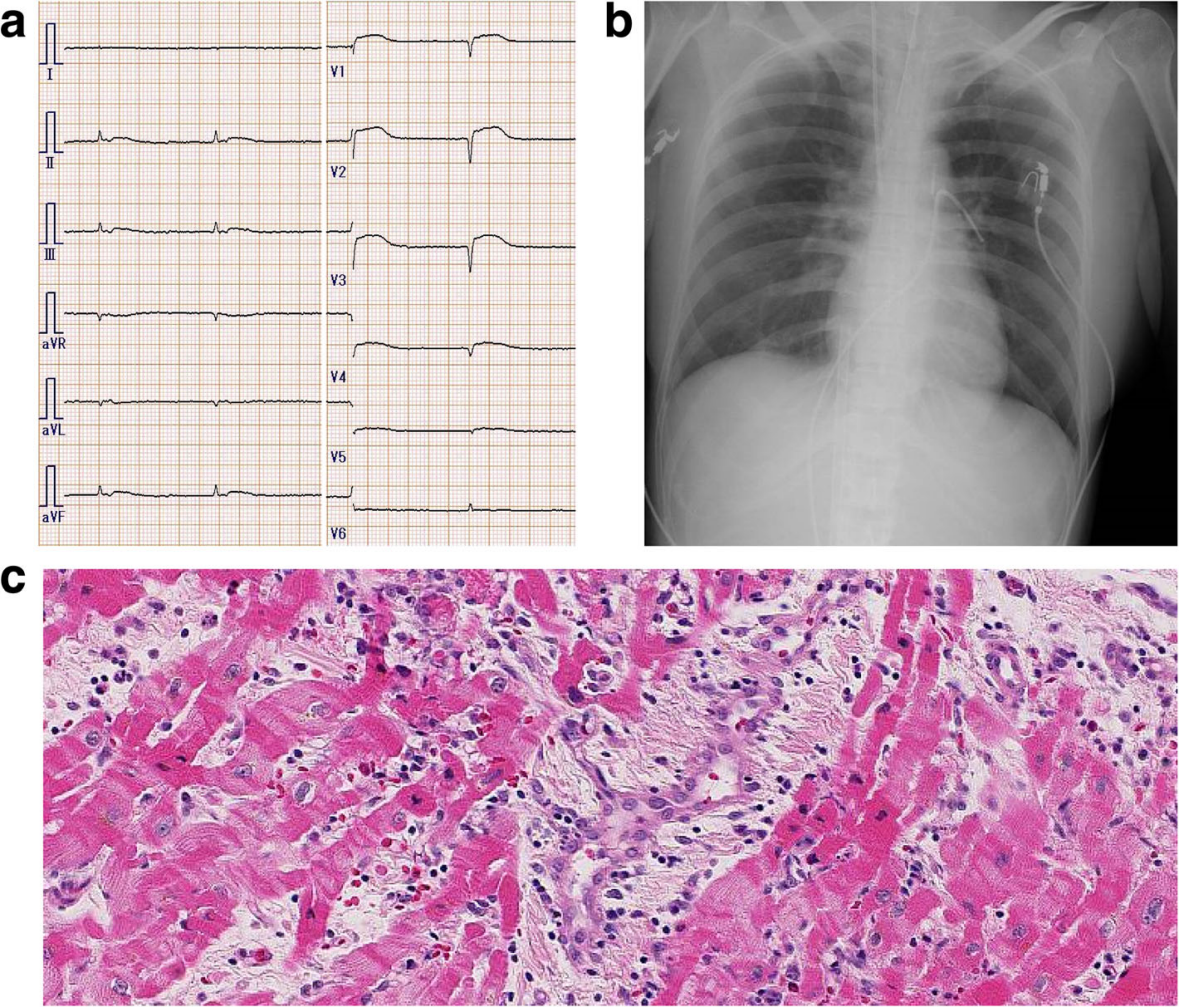
Electrocardiogram and chest X-ray on admission and histopathological findings of cardiac biopsy. Panel **a** Electrocardiogram on admission. Panel **b** Chest X-ray on admission. Panel **c** Hematoxylin-eosin staining of the myocardium obtained from the left ventricular apex: inflammatory cell infiltration composed mainly of lymphocytes, myocardial damage, myocardial disarray, interstitial edema, degeneration of myocardial cells, and the appearance of a contraction band. There were a few eosinophils and neutrophils without evidence of giant cells


Her Glasgow Coma Scale was 9 (E3VTM5), her pupils were 2 mm in diameter, and the bilateral light reflex was prompt. A physical examination revealed peripheral edema and coldness. Her mean blood pressure on admission to our hospital was 60 mmHg with the support of VA-ECMO, IABP, and intravenously administered noradrenaline (0.5 μg/kg per minute). The flow rate of ECMO was 2.8 L/minute (body surface area = 1.50 m^2^). The central venous pressure (CVP) was 15 mmHg regardless of right atrial venous drainage. Laboratory data on admission showed an aspartate aminotransferase (AST) level of 369 U/L, alanine aminotransferase (ALT) level of 212 U/L, serum total bilirubin (T-Bil) level of 1.0 mg/dL, creatine kinase (CK) level of 7537 U/L, creatine kinase-MB (CK-MB) level of 87 U/L, and serum creatinine level of 0.38 mg/dL. An ECG showed an atrioventricular junctional rhythm, a heart rate of 51 beats/minute, and ST elevation in broad precordial leads with low voltage (Fig. 1, Panel a). A chest X-ray showed no remarkable cardiac dilatation or pulmonary congestion (Fig. 1, Panel b). Transthoracic echocardiography on the day after admission (day 4) revealed that her LVEF decreased to 5% and that an opening of the aortic valve was not observed (Additional file 3: Movie 3).

Additional file 3: Movie 3. Transthoracic echocardiography on day 4 revealed that the patient's left ventricular ejection fraction decreased to 5% and that an opening of the aortic valve was not observed. (WMV 255 kb)


**Fig. 2:**
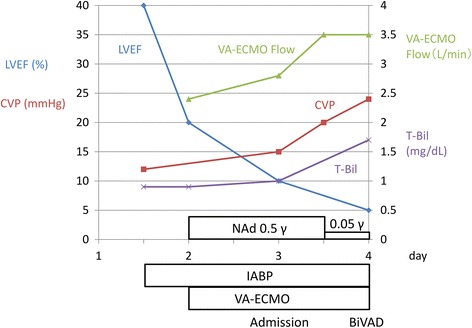
Clinical course before biventricular assist device implantation. *BiVAD* biventricular assist device, *CVP* central venous pressure, *γ* μg/kg per minute, *IABP* intra-aortic balloon pump, *LVEF* left ventricular ejection fraction, *NAd* noradrenaline, *T-Bil* total bilirubin, *VA-ECMO* venoarterial extracorporeal membrane oxygenation

**Fig. 3:**
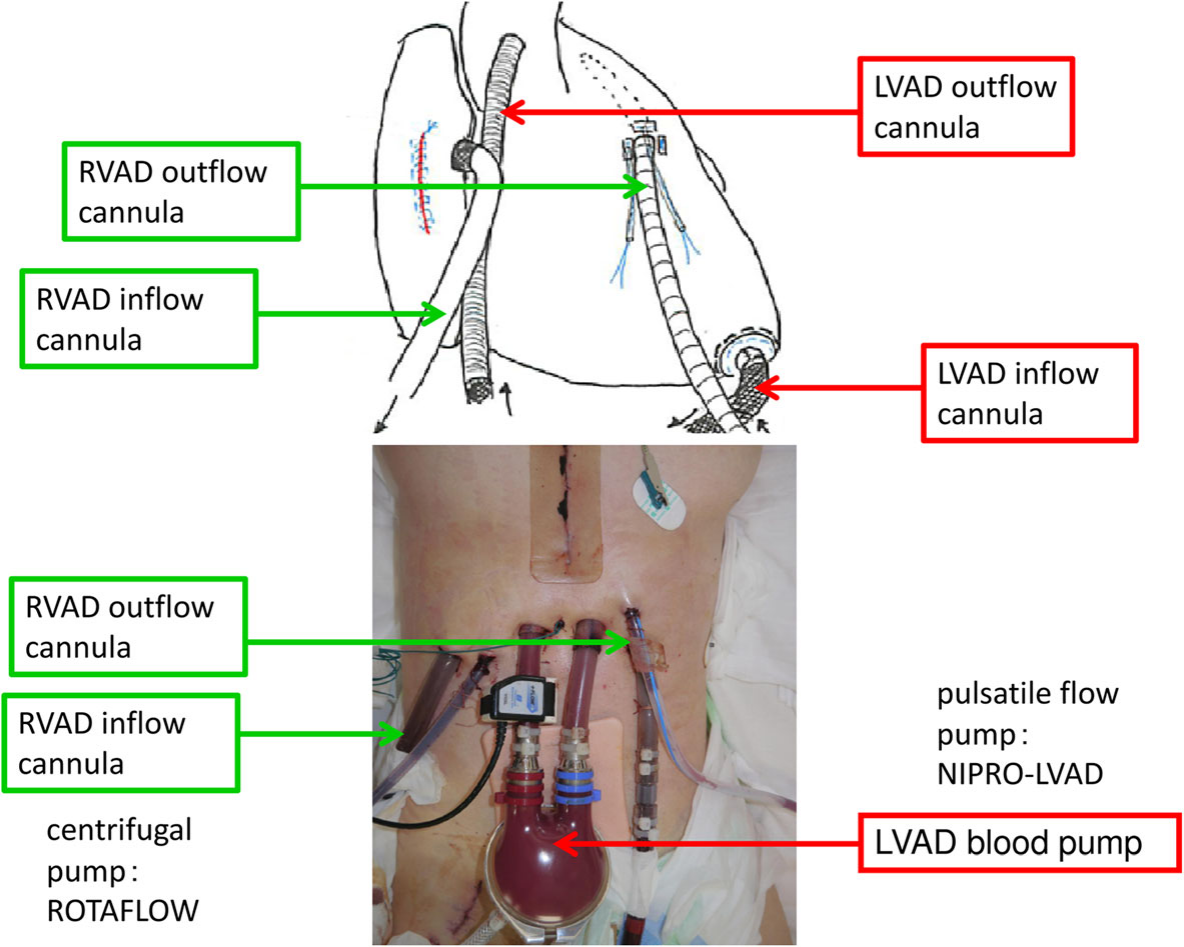
Images after biventricular assist device implantation. We implanted a left ventricular assist device using NIPRO-VAD. We added a right ventricular assist device using ROTAFLOW. *LVAD* left ventricular assist device, *RVAD* right ventricular assist device

The clinical course of our patient is shown in Fig. 2. After admission to our hospital, we increased the VA-ECMO flow from 2.8 to 3.5 L/minute and intravenously administered noradrenaline was successfully reduced from 0.5 to 0.05 μg/kg per minute. However, her CVP continued to elevate from 15 to 24 mmHg accompanied by elevated T-Bil (1.7 mg/dL). A progression of biventricular failure was suspected, and a BiVAD implantation was performed on the next day (day 4). First, we implanted a NIPRO-VAD (NIPRO Co., Osaka, Japan) [2], a paracorporeal pulsatile flow pump, as a left ventricular assist device (LVAD) with an inflow from the left ventricular apex and an outflow to her ascending aorta. When we tried to wean her from cardiopulmonary bypass, a transesophageal echocardiography showed a small left ventricular size with an insufficient filling of the LVAD blood pump and high CVP (15 to 20 mmHg), implying that sufficient blood flow did not circulate to the left side of her heart because of right-sided heart failure. Second, we added a right ventricular assist device (RVAD) using ROTAFLOW (Maquet Japan Co., Tokyo, Japan) [2], a paracorporeal centrifugal pump. An inflow cannula was inserted into her right atrium, and an outflow cannula was inserted through the right ventricular outflow tract to her main pulmonary artery (Fig. 3). The IABP was removed, and intravenously administered noradrenaline was stopped after the VAD implantation with 5 μg/kg per minute intravenously administered dopamine hydrochloride (DOA), 5 μg/kg per minute dobutamine hydrochloride (DOB), 0.25 μg/kg per minute olprinone hydrochloride hydrate, 0.1 μg/kg per minute carperitide (hANP), and 0.5 μg/kg per minute nitroglycerin (NTG). Intravenously administered immunoglobulin therapy (5 g/day) and intravenously administered pulse corticosteroid therapy (methylprednisolone 1 g/day) were administered on days 3 to 5. Her renal function was preserved throughout this clinical course.

A histopathological examination of her myocardium obtained from the left ventricular apex during a VAD operation was consistent with lymphocytic myocarditis: inflammatory cell infiltration composed mainly of lymphocytes, myocardial damage, myocardial disarray, interstitial edema, degeneration of myocardial cells, and the appearance of a contraction band. There were a few eosinophils and neutrophils without evidence of giant cells (Fig. 1, Panel c, hematoxylin-eosin staining). The right ventricular endomyocardial biopsy findings of the local hospital were also consistent with lymphocytic myocarditis. Serological verification from paired samples showed no significant increase in serum viral antibody titers.

The transition of left ventricular systolic function was as follows: before VAD implantation on day 4, her LVEF was below 5% without opening of the aortic valve (Additional file 3: Movie 3). However, her LVEF improved to 40% on the third day after the VAD operation (day 7; Additional file 4: Movie 4). Her LVEF improved to 50% on day 6 after the operation with a complete opening of the aortic valve in every beat (day 10; Additional file 5: Movie 5).


Additional file 4: Movie 4. Transthoracic echocardiography on day 7 revealed the patient's left ventricular ejection fraction improved to 40%. (WMV 115 kb)



Additional file 5: Movie 5. Transthoracic echocardiography on day 10 revealed the patient's left ventricular ejection fraction improved to 50% with a complete opening of the aortic valve in every beat. (WMV 107 kb)


**Fig. 4:**
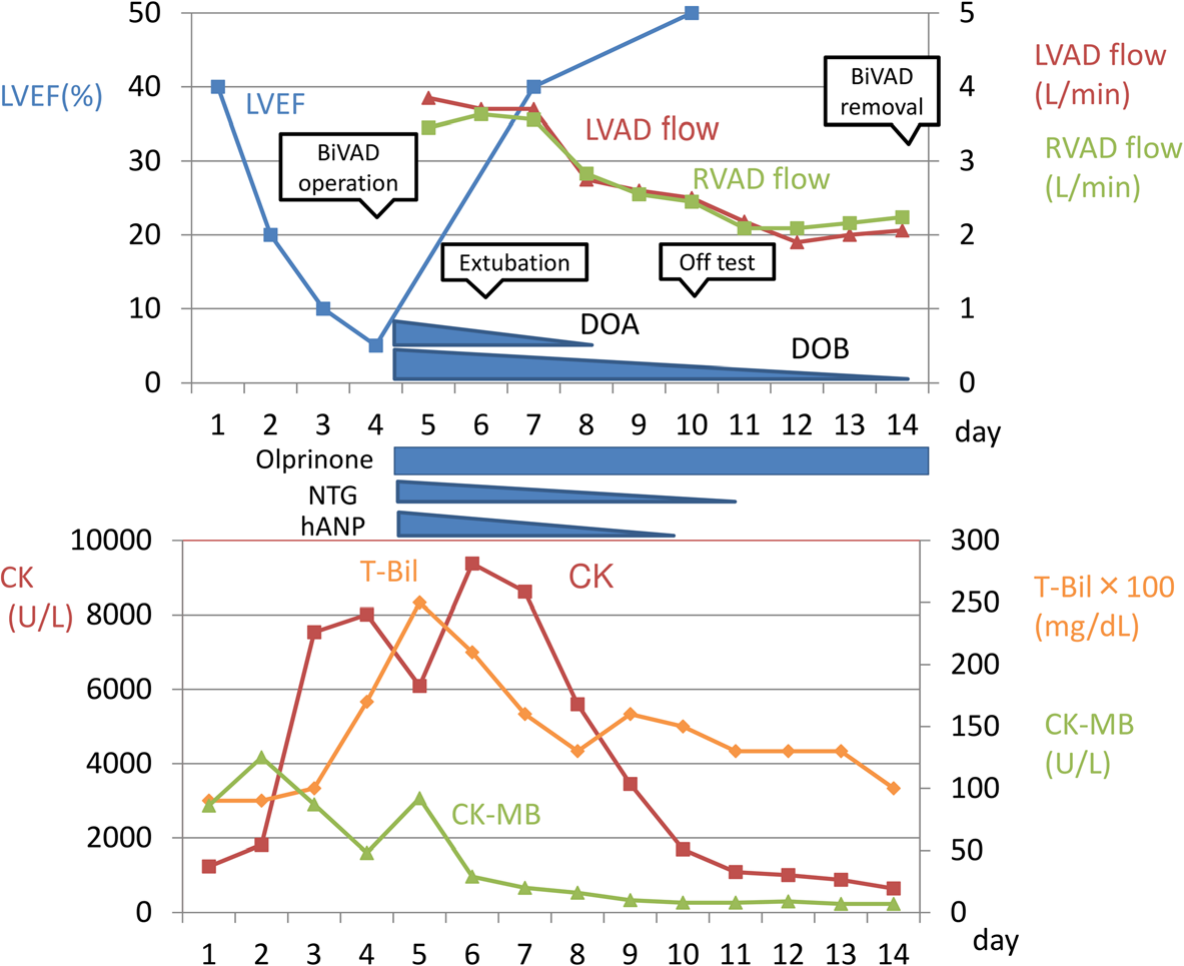
Clinical course after ventricular assist device implantation. The serum total bilirubin level peaked on day 6. The left ventricular ejection fraction improved to 50% on day 10. The biventricular assist device was successfully removed on day 14. *BiVAD* biventricular assist device, *CK* creatine kinase, *CK-MB* creatine kinase-MB, *DOA* dopamine hydrochloride, *DOB* dobutamine hydrochloride, *hANP* carperitide, *LVAD* left ventricular assist device, *LVEF* left ventricular ejection fraction *NTG* nitroglycerin, *Olprinone* olprinone hydrochloride hydrate, *RVAD* right ventricular assist device, *T-Bil* total bilirubin

The time course after the VAD implantation is shown in Fig. 4. Her T-Bil peaked soon after the VAD implantation on day 6 (peak T-Bil level = 2.6 mg/dL). She was extubated on day 6 and started oral intake from day 7. Her LVEF began to recover, and CK peaked on day 7 (peak CK level = 9376 U/L). The LVAD and RVAD flow gradually decreased, reflecting the recovery of native cardiac function. On day 10, we performed a BiVAD-off test with bedside echocardiography. The VAD flow before the test consisted of a 2.5 L/minute RVAD flow and 2.5 L/minute LVAD flow. Intravenous inotropes and vasodilators before the test included 2 μg/kg per minute DOB, 0.25 μg/kg per minute olprinone, and 0.5 μg/kg per minute NTG. We switched off the RVAD and then the LVAD after heparinization, and evaluated the left ventricular diameter on the BiVAD, LVAD, and without VAD support. The left ventricular diastolic diameter (LVDd), left ventricular systolic diameter (LVDs), and LVEF in each condition were as follows: LVDd/Ds = 43/32 mm and LVEF = 50% on BiVAD, 43/28 mm and 66% on LVAD, 44/27 mm and 69% without VAD support. The LVDd did not decrease even after the RVAD was switched off; implying that her right-sided heart function had recovered. The LVDd did not increase markedly after the LVAD was switched off, implying that her left ventricular function had recovered. No significant change of systemic blood pressure was observed during this process.

We performed a BiVAD removal operation on day 14 without a cardiopulmonary bypass. First, we switched off the RVAD after the redo median sternotomy. Intra-arterial blood pressure monitoring and a Swan-Ganz catheter revealed that her mean systemic blood pressure was 81 mmHg, mean right atrial pressure (RAP) was 11 mmHg, and the mean pulmonary artery pressure (PAP) was 19 mmHg without RVAD support. A close observation showed no significant changes for 10 minutes. Thus we removed the RVAD. Second, we performed a LVAD-off test. The mean systemic blood pressure was 69 mmHg, mean RAP was 8 mmHg, mean PAP was 20 mmHg, mean pulmonary artery wedge pressure (PAWP) was 14 mmHg, and cardiac index (CI) was 3.1 L/minute/m^2^ without LVAD support. Her hemodynamics did not show significant changes for 30 minutes, and the LVAD was removed. Echocardiography 12 days after the BiVAD removal (day 26) showed a recovery of cardiac function: LVDd/Ds = 40/26 mm and LVEF = 64%. She was discharged on day 32. During follow-up, she did not have worsening biventricular function over 4 years.

## Discussion

Patients with fulminant myocarditis have a clinical course that is distinct from patients with acute (nonfulminant) myocarditis [3]. McCarthy *et al.* studied 147 patients considered to have myocarditis according to endomyocardial biopsy results and reported that 10.2% (15/147) of the patients had fulminant myocarditis and 89.8% (132/147) had acute myocarditis [3]. Felker *et al.* studied echocardiographic findings in fulminant and acute myocarditis and reported that patients with fulminant myocarditis exhibit a substantial improvement in ventricular function at 6 months compared with those with acute myocarditis [4].

Although patients with fulminant myocarditis are critically ill at presentation, long-term survival is excellent [3]. Whereas, patients with acute myocarditis are less ill initially but have a progressive course including death or the need for cardiac transplantation. Considering the long-term prognosis of patients with fulminant myocarditis, an aggressive approach that includes mechanical circulatory assistance is warranted [3].

Although the results of the VA-ECMO have been excellent in selected patients [5], continued support by VA-ECMO may be associated with significant morbidity. If there is no improvement in cardiac function, the patients should be bridged to VAD therapy. First, VA-ECMO does not unload the left ventricle and is associated with increased left ventricular wall stress [6]. VAD seems to be advantageous from the standpoint of left ventricular load reduction and recovery of cardiac function. Second, VA-ECMO is sometimes not able to provide sufficient blood perfusion because of a small cannula and severe hemolysis [1]. Although we increased the VA-ECMO flow after admission, CVP continued to increase accompanied by elevated T-Bil, implying the exacerbation of right-sided heart failure. VAD is a better device for prevention and improvement of multiple organ failure including liver failure. In patients with LVAD or BiVAD support, preoperative T-Bil levels were identified as a predictive factor of patient survival [7, 8]. The VA-ECMO should be changed to VAD before the bilirubin level increases [7, 9]. Third, we can provide rehabilitation to patients under the support of BiVAD. In the present case, our patient was extubated 2 days after the operation and started oral intake in the sitting position from the third day. Fourth, peripheral vascular complications concerning VA-ECMO such as lower extremity ischemia are not negligible [1]. Accordingly, VAD therapy should be considered if there is no improvement in cardiac function regardless of several days of VA-ECMO support [10] before the bilirubin level increase.

Biventricular involvement is not uncommon in fulminant myocarditis. Over 70% of right-sided heart involvement has previously been reported in patients with fulminant myocarditis [11]. Therefore, RVAD should be added when there are signs of right ventricular failure after LVAD implantation.

## Conclusions

For treatment of myocarditis with VA-ECMO, the possibility of initiation of VADs should be considered. RVAD implantation is indicated in cases accompanied with severe right ventricular dysfunction. RVAD should be added when necessary because biventricular involvement is not uncommon in fulminant myocarditis.

## Abbreviations

ALT: Alanine aminotransferase
AST: Aspartate aminotransferase
BiVAD: Biventricular assist device
CI: Cardiac index
CK: Creatine kinase
CK-MB: Creatine kinase-MB
CVP: Central venous pressure
DOA: Dopamine hydrochloride
DOB: Dobutamine hydrochloride
ECG: Electrocardiogram
IABP: Intra-aortic balloon pump
LVAD: Left ventricular assist device
LVDd: Left ventricular diastolic diameter
LVDs: Left ventricular systolic diameter
LVEF: Left ventricular ejection fraction
NTG: Nitroglycerin
PAP: Pulmonary artery pressure
PAWP: Pulmonary artery wedge pressure
RAP: Right atrial pressure
RVAD: Right ventricular assist device
T-Bil: Total bilirubin
VAD: Ventricular assist device
VA-ECMO: Venoarterial extracorporeal membrane oxygenation
